# Occipital Lobe Epilepsy With Ictal Fear: Evidence From a Stereo-Electroencephalography (sEEG) Case

**DOI:** 10.3389/fneur.2018.00644

**Published:** 2018-08-07

**Authors:** Jing Wang, Qian Wang, Mengyang Wang, Guoming Luan, Jian Zhou, Yuguang Guan, Zhaofen Yan

**Affiliations:** ^1^Department of Neurology, Sanbo Brain Hospital, Capital Medical University, Beijing, China; ^2^Department of Clinical Psychology, Sanbo Brain Hospital, Capital Medical University, Beijing, China; ^3^Department of Functional Neurosurgery, Sanbo Brain Hospital, Capital Medical University, Beijing, China; ^4^Beijing Key Laboratory of Epilepsy, Epilepsy Center, Sanbo Brain Hospital, Capital Medical University, Beijing, China; ^5^Beijing Institute for Brain Disorders, Beijing, China

**Keywords:** stereo-electroencephalography (sEEG), epilepsy, ictal fear, occipital cortex, focal cortical dysplasia (FCD)

## Abstract

Ictal fear—a relatively rare phenomenon—is a semiological characteristic of epilepsy. Most patients with epilepsy with ictal fear have an epileptic zone in the mesial temporal lobe, which is the classical brain area involved in emotion processing. Herein, we report a case of epilepsy with ictal fear as the first manifestation in a 10-year-old boy. All noninvasive evaluation including scalp video electroencephalography (EEG), magnetic resonance imaging (MRI), and positron emission tomography/computed tomography (PET-CT) suggested a possible lesion in the left posterior brain region. Stereo-electroencephalography (sEEG) results showed high frequency direct current shift in the left occipital lobe 1 s before the fear manifestation which preceded in 12 s the discharge in the amygdala. This case highlights the epileptic network hypothesis which suggested occipital cortex may play an important role in the early emotional network independently of amygdala activation.

## Background

Ictal fear is a relatively rare symptom in epileptic seizures (1–4). Present understanding suggests that fear appearing at the early stage of a clinical seizure is one of the characteristics that involves the orbital frontal cortex (OFC), anterior cingulate, and temporal limbic cortices network (5). The amygdala has been proven to play a crucial role in epileptic fear (6–8). Accumulating cognitive experiments have shown that the amygdala processes threatening information like fear or anger (9, 10).

Oehl et al. (11) have reported that an epileptic patient with ictal fear originating from the right occipital lobe that was ahead of the discharge in the amygdala, which is the only intracranial electroencephalography (EEG) evidence to our best knowledge. However, whether occipital cortex is involved in the emotional processing is still debatable (12).

Herein, using stereo-electroencephalography (sEEG), we report a rare case of epileptic seizures with lack of activation of amygdala during ictal fear, and further confirm the onset of the seizure in the left posterior brain areas by simultaneously recording the amygdala and occipital cortex.

## Case presentation

The patient was a 10-year-old right-handed boy. The duration of the illness was 3 years. The first episode occurred at the age of 7 years, without obvious inducement. Symptomatic manifestations included sudden shouting, right-side strabismus, salivation, and generalized tonic-clonic seizures (GTCS), which lasted for about 4–5 min. The second episode occurred 10 days later, with similar symptomatic manifestations. There was no seizure for 1 year after oral oxcarbazepine medication. Seizures then occurred at the age of 8 years, with symptomatic manifestations such as panic, shouting, and tachycardia and lasted about 1–2 min without loss of consciousness. Headache, left eye pain, abdominal pain, and nausea would occasionally appear in the post-ictal period. Gradually, the patient's seizure frequency ranged from once a month to 4 times/day. Oral administration of oxcarbazepine was ineffective. History of perinatal hypoxia, febrile convulsions, brain injury, and family history of epilepsy were negative. Physical examination showed stable vital signs, and the results of neurological examination were normal. Blood routine, biochemical tests, infection immunoassay, blood coagulation tests, and urinalysis were all normal. Electrocardiogram and chest radiography showed normal results.

**Figure 1:**
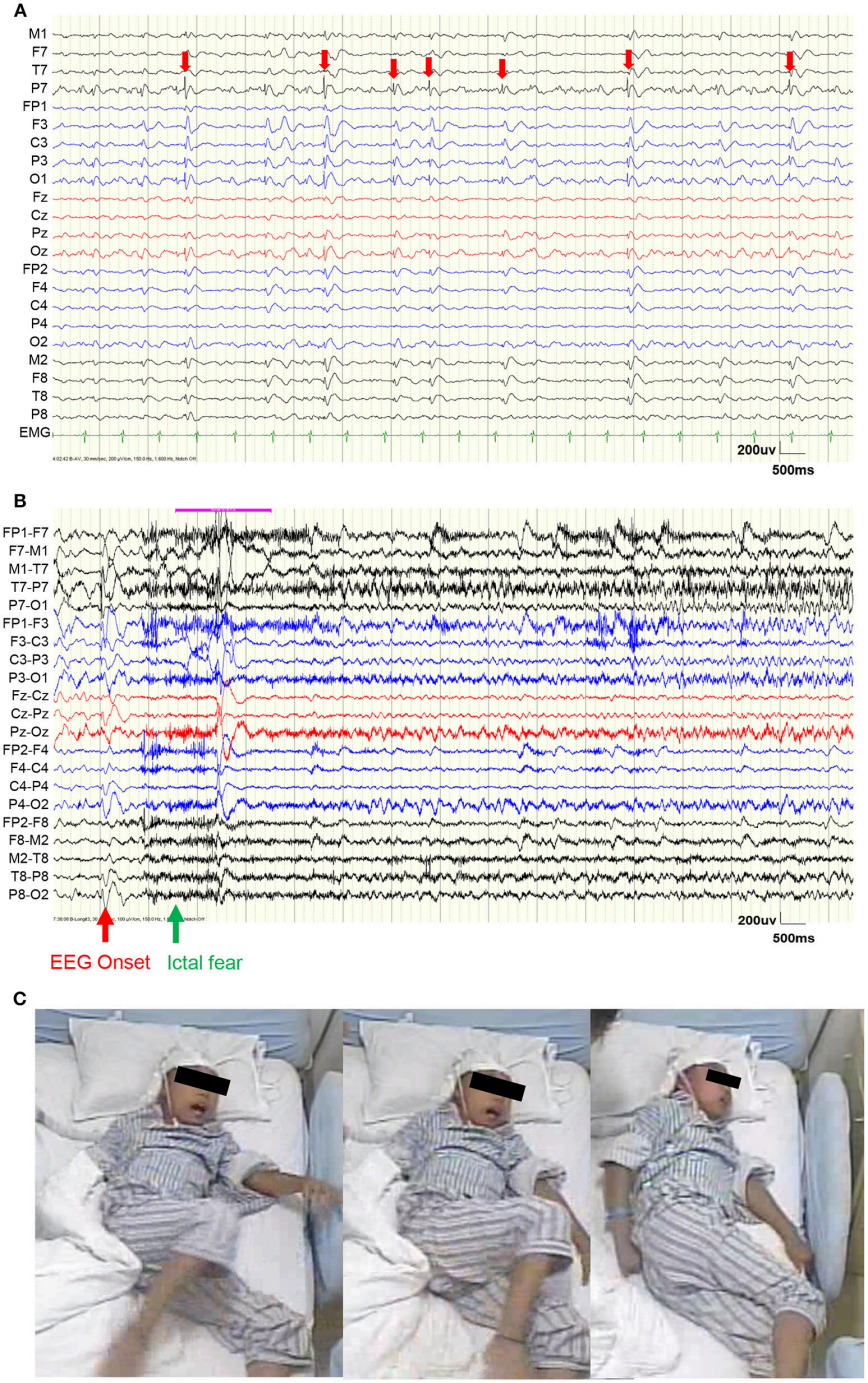
Video EEG results. **(A)** EEG during a representative interictal period. Red arrows show the interictal epileptiform discharges (IEDs), which were mainly distributed within the left occipital area. **(B)** EEG before a representative seizure. The red arrow shows the EEG onset and the green arrow shows the ictal fear. **(C)** Semiology of ictal fear.

To evaluate the ictal fear, both video observation and neurologic interview were conducted according to the diagnosis criteria of ictal fear (13). First of all, fearful facial expression and screaming had been observed before the clinical seizure (Figure 1C). The content of screaming includes “Aha! Aha!” or “Mama!” or “Mama! Find the doctor!” Secondly, the patient could remember and describe feelings of fear, without any specific content or scene. Visual aura or other associated aura was denied. The last but not the least, the patient described this fear feeling start abruptly, concomitant with the seizure.

Scalp video EEG (vEEG) were recorded using a Nicolet video-EEG monitoring system (Thermo Nicolet Corporation, USA), digitized at the rate of 1,024 Hz with the international standard 10–10 electrodes montage. The on-line band-pass filter was 1.6–150 Hz. The patient was monitored for a total of 6 days. During the monitoring, oxcarbazepine was gradually reduced and one typical epileptic seizure was captured (Figure 1).

**Figure 2:**
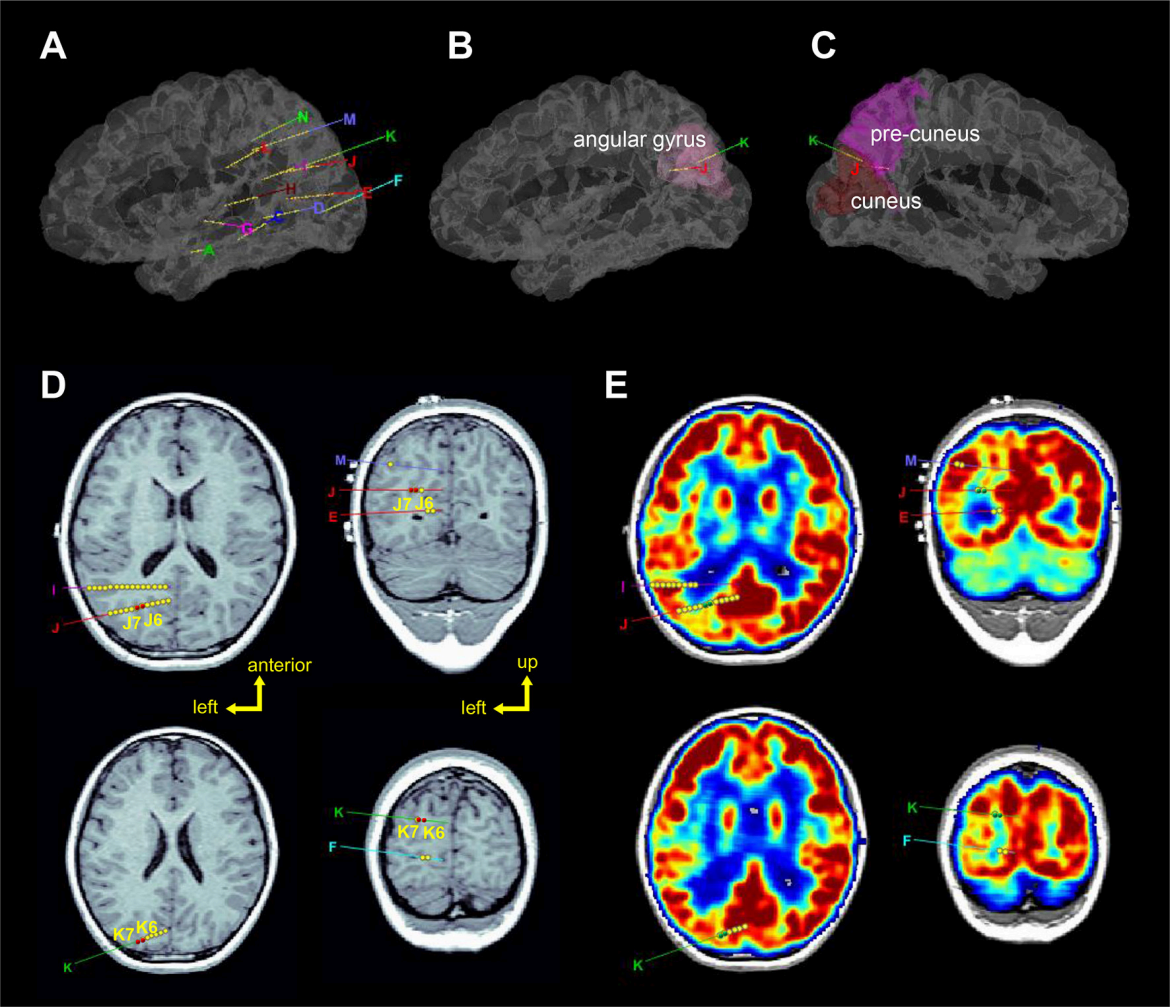
Localization of the sEEG electrodes (left hemisphere). **(A–C)** Represent 3D views of the electrode locations in the stereotactic space, in the brain model reconstructed from individual T1-image of the patients. Electrodes J and K were entered from the angular gyrus and ended at the precuneus and cuneus, respectively. **(D)** Axial and coronal views of electrodes J and K superimposed on T1-image. Red circles (J6, J7, K6, and K7) show the seizure-onset zones which were located in the sulci. **(E)** Axial and coronal views of electrodes J and K superimposed on PET-image which was registered to the T1-image. Green circles (J6, J7, K6, and K7) show the seizure-onset zones which were located in low metabolism regions.

Figure 1A shows the vEEG waveforms during the interictal period. Interictal epileptiform discharges (IEDs) could be observed (denoted by red arrows), which were mainly distributed within the left occipital electrodes (P7 and O1). Figure 2B shows the vEEG waveforms before the epileptic seizure. The EEG onset occurred 1.5 s earlier than the ictal fear. Fearful facial expression and screaming could be observed in the video (Figure 1C).

Brain imaging results showed that while the T2 signals within the left posterior temporal, occipital, and parietal lobes were high, the T2 signals in the left hippocampus was higher (Figure 2). Further positron emission tomography/computed tomography (PET-CT) examination showed that low metabolic areas were mainly distributed in the left posterior region (Figure 2E).

Stereotactic electrodes were intracranially implanted for further epileptic zone evaluation. A robot-assisted stereotactic operation system (ROSA) was used to place 13 electrodes. The sEEG electrodes were manufactured by Huake Hengsheng Medical Technology Co Ltd., Beijing, China. The diameter of a depth electrode was 0.8 mm. The length of each node was 2 mm, which were spaced 1.5-mm apart from each other. The reference electrode was placed on the forehead. On recording days, the impedance of all the recording electrode nodes was kept below 50 kΩ, and the nodes whose impedances were higher than this value were excluded from analyses. Video EEG was monitored for 6 days after implantation, and the oxcarbazepine dosage was gradually reduced during the monitoring; during this period, a total of 4 seizures were captured. Three-dimensional brain images were reconstructed by pre-implantation of T1 images using BrainSuite (Version 18a, http://brainsuite.org/). Pre-implantation PET-CT and post-implantation CT (included electrode localizations) were co-registered to pre-implantation T1 images using BioImage software (http://bioimagesuite.yale.edu). The localization of sEEG electrodes were then presented using the Brainstorm toolbox [(14); http://neuroimage.usc.edu/brainstorm/] in the MATLAB environment (Figure 2).

**Figure 3:**
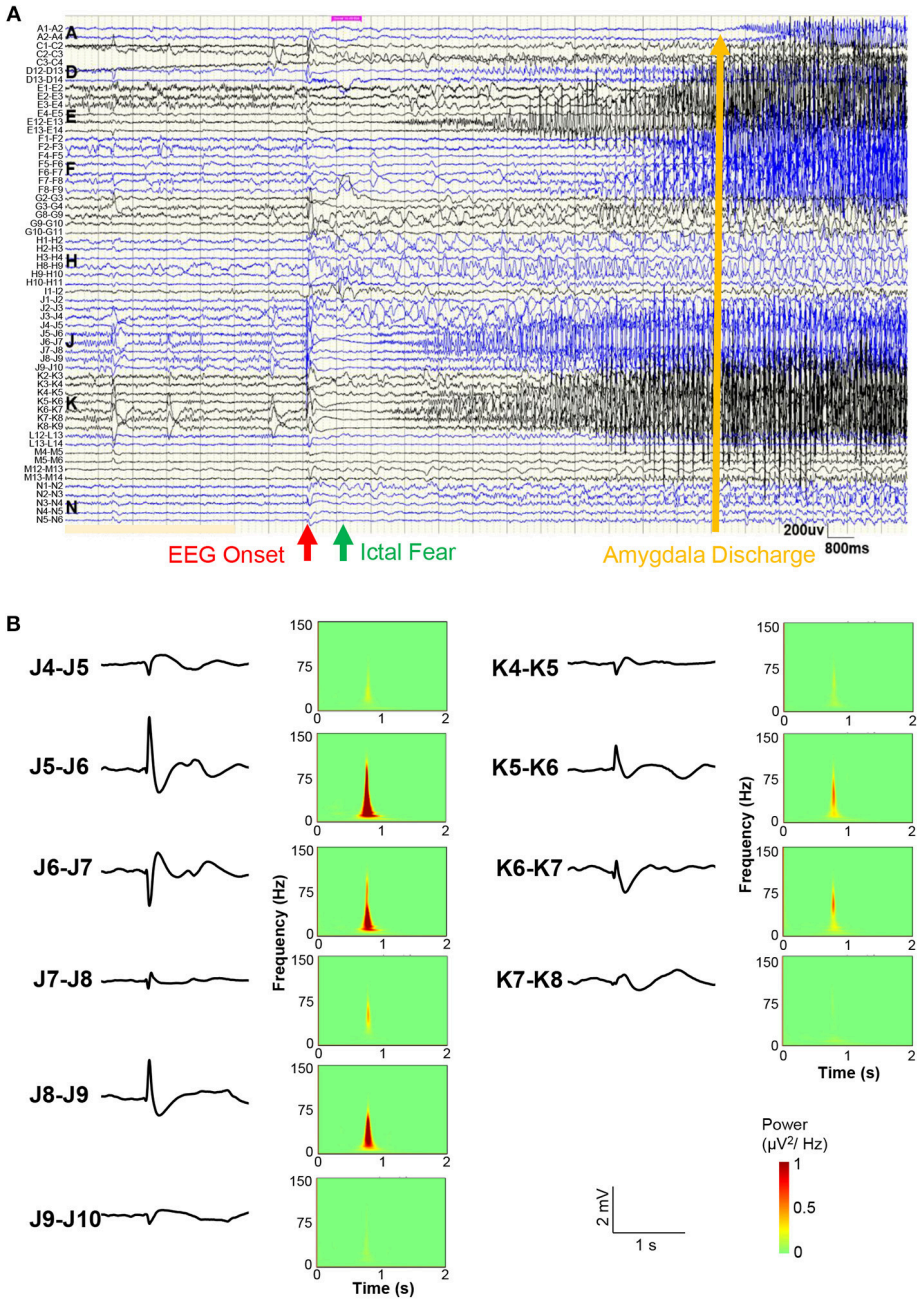
Stereo-EEG results. **(A)** Bipolar lead view of intracranial EEG before a representative seizure. The red arrow shows the EEG onset, the green arrow shows the ictal fear, and the yellow arrow shows the amygdala (A1-A2) discharge time. **(B)** Waveforms and power spectra of EEG onset in electrodes J and K. Simultaneous direct current (DC) drifts were observed in J5-J6, J6-J7, J8-J9, K5-K6, and K6-K7 (occipital lobe).

Figure 3 shows the sEEG results before a representative seizure. A bipolar lead view showed that the EEG onset was 1 s before ictal fear and 12 s before amygdala (A1-A2) and posterior hippocampus (C2-C3) (Figure 3A). At the moment of the start of EEG, simultaneous direct current (DC) drifts were observed in J5-J6, J6-J7, J8-J9, K5-K6, and K6-K7, with high frequency components in spectra (Figure 3B). Direct electrical stimulation (frequency = 50 Hz; pulse wide = 0.3 ms; duration = 5 s; intensity = 1–6 mA) confirmed that only stimulation on J8-J9 could evoked a clinical seizure.

**Figure 4:**
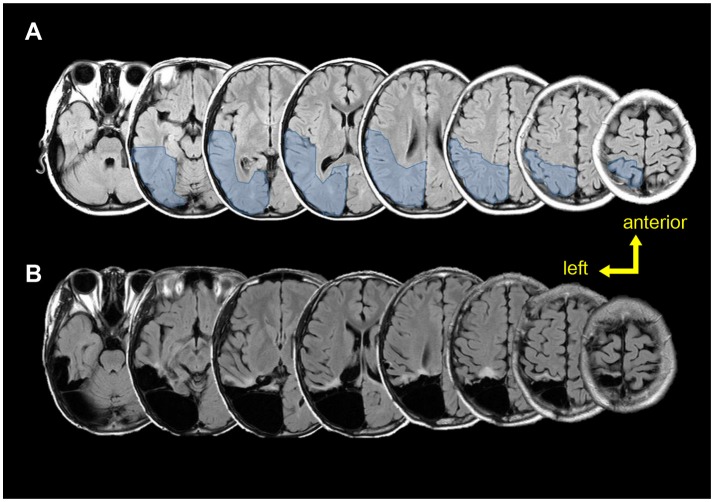
Pre-post-surgical T2 flair magnetic resonance images. **(A)** Pre-surgical images. Light blue region shows the planned resection range. **(B)** Post-surgical (12 months after surgery) images show the true resection range.

According to the sEEG results, the epileptic zone was mainly distributed in the right posterior parietal, posterior temporal, and occipital areas (Figure 4), and then propagated to the posterior part of the middle and inferior temporal gyrus, the posterior part of the hippocampus, the anterior part of the lingual gyrus occipital tongue, and the anterior part of the precuneus. Combined with the evidences of brain imaging (T2 flair and PET-CT), the resection range was planned as shown in Figures 4A,**B** shows the actual lesion area a year after surgery. The pathological examinations showed focal cortical dysplasia (FCD) type 2A in the right parietal-occipital area.

According to the follow-up, the neuropsychological performance before and after the surgery were all in the normal range (Table 1). No seizure occurred after surgery.

**Table 1:** Neuropsychological scores before and after epileptic zone resection.

		**Post-surgery**		
	**Pre-surgery**	**3 months**	**6 months**	**12 months**
FIQ	109	93	105	121
VIQ	111	106	129	142
PIQ	106	98	104	110
MQ	96	95	106	110

*FIQ, full intelligence quotient; VIQ, verbal intelligence quotient; PIQ, performance intelligence quotient; MQ, memory quotient*.

## Discussion

The patient's symptomatology was characterized by ictal fear as the first manifestation. The results of scalp EEG indicated that the onset of epileptiform discharge localized in the bilateral posterior head, which was significantly larger in the left occipital, parietal, and posterior temporal areas characterized by the 6–7 Hz oscillation. The clinical symptoms appeared 1.5 s after the EEG onset, while the frontal and temporal electrodes did not have epileptic discharges at the beginning of the clinical seizure. In the later stage, the left fronto-temporal electrodes showed the 3.5–4 Hz oscillation. Therefore, the scalp vEEG results indicated that the seizures may have originated from the left occipital, parietal, and posterior temporal areas. The brain imaging evidences (T2 flair and PET-CT) also suggest the possible pathological changes in the left occipital, parietal, and posterior temporal areas. However, the first manifestation was ictal fear, which is unusual for episodes of seizure originating from posterior brain areas. It is reported that ictal fear is not only associated with the epileptic discharges in the amygdala, but also occur when the anterior cingulate cortex, orbital frontal cortex, and temporal lobes are hyperactive (5). Under pathological conditions, the inhibition from the amygdala to the OFC is reduced, which causes the release of negative emotions such as fear and anger.

In the current case study, the sEEG recordings of seizure onset shows that high frequency direct current drift occurred in the left occipital cortex. The time interval between onset of discharge and fear symptoms was about 1 s. The early discharge before the symptom involved the neocortex in the posterior part of the temporal and lower temporal gyrus, posterior part of the hippocampus, anterior part of the occipital cortex, and anterior part of the anterior wedge, and the type of discharge mainly manifested as the rhythmic spike-slow/spike waves within α and θ bands. At about 17 s after the EEG onset, the discharge was then involved in the superior temporal gyrus and the amygdala.

How the occipital cortex is involved in emotional processing is still unclear. A previous intracranial EEG study suggested that fear-specific responses are not restricted to the amygdala, rather also occur in the OFC, lateral occipital lobe, lingual gyrus, and anterior temporal lobe (12). In this study, specific responses to fear were first recorded in the amygdala (after 200 ms) and then, in the occipito-temporal visual regions (after 300 ms). This finding implied a possible participation of the occipital cortex in the early networks of fear processing.

Thus far, there are only few reports of ictal fear originating from the posterior brain area. Guimond et al. (8) reviewed 144 cases with ictal fear and found that 11 out of the 144 cases resulted in occipital epileptic focus. First evaluating by intracranial EEG, (11) reported an epileptic patient with ictal fear originating from the right occipital lobe, which was ahead of the discharge in the amygdala regions. Fear was triggered by spontaneous discharge in the occipital lobe and by electrical stimulation at the same location via the secondary propagation of discharges from the occipital epileptogenic area to the amygdala. To our best knowledge, our case report is only the second to show the occipital origins of ictal fear evaluated by intracranial EEG.

Compared with the case reported by Oehl et al. (11), there exist several differences. First, Oehl et al. reported a right occipital focus while the current case reports a left occipital focus. According to the literature, among patients with ictal fear symptoms, 68% have a right hemisphere focus and 32% have a left hemisphere focus (8). Second, Oehl et al. used subdural electrodes to monitor the lateral gyrus of the occipital cortex, whereas we conducted sEEG to monitor the sulcus of the occipital cortex. Lastly, Oehl's case focused on non-epileptic disorder, while our case report did not.

Interestingly, in the current case, the ictal activity started 1 s before fear reducing the possibility of symptoms coming from other non-sampled brain areas. Moreover, it occurs 12 s before the propagation to amygdala. This support the two-system framework of fear, that regions other than amygdala, including occipital cortex, also participate in the “cognitive circuit” which related to the self-report feelings to fear (15). The presence of cortical dysplasia taken together with the 12 months period of seizure free showed in this case report support the idea the epileptogenic zone was included in the left posterior cortex.

In conclusion, using sEEG technology, we report in this case study that epilepsy with ictal fear as the first symptom could originate from the left occipital cortex. We believe this report will help to form the epileptic network hypothesis, suggesting that the epileptic zone of the ictal fear should not be considered only in the amygdala.

## Ethics statement

This study was carried out in accordance with the recommendations of the Regional Ethics Committee of Sanbo Brain Hospital. As the patient is a minor, written informed consent from his parents were provided including the permission for scientific publication. The protocol was approved by the Ethics Committee of Sanbo Brain Hospital, Capital Medical University.

## Author contributions

JW and QW collected patient information, conceptualized and designed the study, and drafted the manuscript. MW and ZY clinical data analysis. GL, JZ, and YG electrode implantation and lobectomy implementation.

### Conflict of interest statement

The authors declare that the research was conducted in the absence of any commercial or financial relationships that could be construed as a potential conflict of interest.

## References

[1] Alemayehu S Bergey GK Barry E Krumholz A Wolf A Fleming CP. Panic attacks as ictal manifestations of parietal lobe seizures. Epilepsia (1995) 36:824–30. 7635102 10.1111/j.1528-1157.1995.tb01621.x

[2] Paparrigopoulos T Kyrozis A Tzavellas E Karaiskos D Liappas I. Left parieto-occipital lesion with epilepsy mimicking panic disorder. Prog Neuro-psychoph. (2008) 32:1606–8. 10.1016/j.pnpbp.2008.04.015 18538459

[3] Kuzniecky R. Symptomatic occipital lobe epilepsy. Epilepsia (1998) 39:24–31. 10.1111/j.1528-1157.1998.tb05122.x 9637590

[4] Taylor I Scheffer IE Berkovic SF. Occipital epilepsies: identification of specific and newly recognized syndromes. Brain (2003) 126:753–69. 10.1093/brain/awg080 12615636

[5] Biraben A Taussig D Thomas P Even C Vignal JP Scarabin JM. Fear as the main feature of epileptic seizures. J Neurol Neurosurg Psychiatry (2001) 70:186–91. 10.1136/jnnp.70.2.186 11160466 PMC1737203

[6] Wieser HG. Mesial temporal lobe epilepsy versus amygdalar epilepsy: late seizure recurrence after initially amygdalotomy and regained seizure control following hippocampectomy. Epileptic Disord. (2000) 2:141–52. 11022139

[7] Blair HT Huynh VK Vaz VT Van J Patel RR Hiteshi AK. Unilateral storage of fear memories by the amygdala. J Neurosci. (2005) 25:4198–205. 10.1523/JNEUROSCI.0674-05.2005 15843623 PMC6724944

[8] Guimond A Braun CM Belanger E Rouleau I. Ictal fear depends on the cerebral laterality of the epileptic activity. Epileptic Disord. (2008) 10:101–12. 10.1684/epd.2008.0184 18539560

[9] Calder A Lawrence A Young A. Neuropsychology of fear and loathing. Nat Rev Neurosci. (2001) 2:352–63. 10.1038/35072584 11331919

[10] Morris JS Frith CD Perrett DI Rowland D Young AW Calder AJ. A differential neural response in the human amygdala to fearful and happy facial expressions. Nature (1996) 383:812–5. 10.1038/383812a0 8893004

[11] Oehl B Schulze-Bonhage A Lanz M Brandt A Altenmüller DM. Occipital lobe epilepsy with fear as leading ictal symptom. Epilepsy Behav. (2012) 23:379–83. 10.1016/j.yebeh.2011.12.014 22373717

[12] Krolak-Salmon P Hénaff MA Vighetto A Bertrand O Mauguière F. Early amygdala reaction to fear spreading in occipital, temporal, and frontal cortex. Neuron (2004) 42:665–76. 10.1016/S0896-6273(04)00264-8 15157426

[13] Leal RB Lopes MW Formolo DA Carvalho CR Hoeller AA Latini A (2018). Amygdala levels of the GluA1 subunit of glutamate receptors and its phosphorylation state at serine 845 in the anterior hippocampus are biomarkers of ictal fear but not anxiety. Mol psychiatry. 10.1038/s41380-018-0084-7. [Epub ahead of print]. 29880883

[14] Tadel F Baillet S Mosher JC Pantazis D Leahy RM. Brainstorm: a user-friendly application for meg/eeg analysis. Comput Intell Neurosci. (2011) 2011:879716. 10.1155/2011/879716 21584256 PMC3090754

[15] Ledoux JE Pine DS. Using neuroscience to help understand fear and anxiety: a two-system framework. Am J Psychiatry (2016) 173:1–11. 10.1176/appi.ajp.2016.16030353 27609244

